# Risk of severe illness of COVID‐19 patients with NAFLD and increased NAFLD fibrosis scores

**DOI:** 10.1002/jcla.23880

**Published:** 2021-07-02

**Authors:** Renling Yao, Li Zhu, Jian Wang, Jiacheng Liu, Ruifei Xue, Leyang Xue, Longgen Liu, Chunyang Li, Haiyan Zhao, Juan Cheng, Songping Huang, Yang Li, Xiang‐an Zhao, Chuanwu Zhu, Ming Li, Rui Huang, Chao Wu

**Affiliations:** ^1^ Department of Infectious Diseases Nanjing Drum Tower Hospital Clinical College of Nanjing Medical University Nanjing China; ^2^ Department of Infectious Diseases Nanjing Drum Tower Hospital The Affiliated Hospital of Nanjing University Medical School Nanjing China; ^3^ Department of Infectious Diseases The Affiliated Infectious Diseases Hospital of Soochow University Suzhou China; ^4^ Department of Infectious Diseases Nanjing Drum Tower Hospital Clinical College of Traditional Chinese and Western Medicine Nanjing University of Chinese Medicine Nanjing China; ^5^ Department of Critical Medicine Huai'an No. 4 People's Hospital Huai’an China; ^6^ Department of Infectious Diseases The Third People's Hospital of Changzhou Changzhou China; ^7^ Department of Infectious Diseases Affiliated Hospital of Xuzhou Medical University Xuzhou China; ^8^ Department of Infectious Diseases The People’s Hospital of Suqian Suqian China; ^9^ Department of Infectious Diseases Yancheng Second People’s Hospital Yancheng China; ^10^ Department of Infectious Diseases Nantong Third People's Hospital Nantong University Nantong China; ^11^ Department of Infectious Diseases Taizhou People's Hospital Taizhou China; ^12^ Department of Gastroenterology Northern Jiangsu People’s Hospital Clinical Medical College of Yangzhou University Yangzhou China; ^13^ Department of Hepatology The Affiliated Infectious Diseases Hospital of Soochow University Suzhou China

**Keywords:** COVID‐19, fibrosis, non‐alcoholic fatty liver disease, severe illness

## Abstract

**Background:**

There is still little knowledge about the association of liver fibrosis with the clinical outcomes of COVID‐19 patients with non‐alcoholic fatty liver disease (NAFLD). The aim of the study was to determine the association of NAFLD fibrosis score (NFS)–determined liver fibrosis with clinical outcomes of COVID‐19 patients with NAFLD.

**Methods:**

The NAFLD was diagnosed by the Hepatic Steatosis Index (HSI) in the absence of other causes of chronic liver diseases. NFS was used to evaluate the severity of liver fibrosis.

**Results:**

A total of 86 COVID‐19 patients with NAFLD were included. The median age was 43.5 years, and 58.1% of patients were male. Thirty‐eight (44.2%) patients had advanced liver fibrosis according to the NFS. Multivariate analysis indicated that concurrent diabetes (odds ratio [OR] 8.264, 95% confidence interval [CI] 1.202–56.830, *p* = 0.032) and advanced liver fibrosis (OR 11.057, 95% CI 1.193–102.439, *p* = 0.034) were independent risk factors of severe illness in COVID‐19 patients with NAFLD.

**Conclusion:**

NAFLD patients with NFS‐determined advanced liver fibrosis are at higher risk of severe COVID‐19.

## Graphical abstract

Concurrent with NAFLD is common in COVID‐19 patients. The presence of advanced liver fibrosis was an independent risk factor of severe illness in COVID‐19 patients with NAFLD.

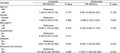

## 1 INTRODUCTION

The coronavirus disease 2019 (COVID‐19), caused by severe acute respiratory syndrome coronavirus 2 (SARS‐CoV‐2), has affected millions of individuals globally. The spectrum of COVID‐19 ranges from asymptomatic disease to critical illness with fatal outcome.^1^ Given the significant impacts of disease severity on prognosis of COVID, much interest has been paid to identify the risk factors associated with severe illness of COVID‐19. Several factors, such as older age, pre‐existing lung disease, diabetes mellitus, cardiovascular disease, and obesity, are associated with the severe illness of COVID‐19.^2^, ^3^, ^4^, ^5^ Chronic liver disease (CLD) is another comorbid condition that is being evaluated for its impact on COVID‐19 severity. It is reported that 2%–11% of patients with COVID‐19 had comorbidities of CLDs.^6^ Non‐alcoholic fatty liver disease (NAFLD) is one of the most common CLDs worldwide.^7^ The global prevalence of NALFD in general population is 6.3%‐45%.^7^, ^8^ In most Asian countries including China, the prevalence of NAFLD is above 25%.^9^

Our previous study demonstrated that 30.7% of COVID‐19 patients had NAFLD.^10^ We also found that COVID‐19 patients with NAFLD are more likely to develop liver injury.^10^ Ji et al. found that COVID‐19 patients with fatty liver had a higher risk of disease progression, higher likelihood of abnormal liver function tests, and longer shedding time of SARS‐CoV‐2 virus.^11^ Mushtaq et al also reported that NAFLD was an independent predictor of liver injury in hospitalized patients with COVID‐19, while NAFLD was not identified as an independent predictor of mortality, disease severity, or disease progression.^12^ NAFLD includes a wide spectrum of CLDs, from steatosis to non‐alcoholic steatohepatitis, with a variable stage of fibrosis to cirrhosis.^13^ The association of severity of liver fibrosis and the COVID‐19 is not yet clear. Targher et al. found that metabolic dysfunction‐associated fatty liver disease (MAFLD) patients with increased FIB‐4 are at higher risk of severe illness of COVID‐19.^14^ The NAFLD fibrosis score (NFS) was demonstrated to identify NAFLD patients with and without advanced fibrosis independently.^15^ Nevertheless, at present, there is still little knowledge about the association of NFS with the severity of COVID‐19 patients with NAFLD. We aimed to determine the association of NFS‐determined liver fibrosis with clinical outcomes of COVID‐19 in NAFLD patients.

## 2 METHODS

### 2.1 Study design and data collection

We retrospectively screened 342 consecutively confirmed COVID‐19 patients who were admitted to hospitals between January 18, 2020, and February 26, 2020, from ten designated hospitals of Jiangsu Province, China. The patients were followed up to February 29, 2020. The COVID‐19 was diagnosed according to the guidance provided by the World Health Organization (WHO).^16^

Patients with viral hepatitis defined by positive serum hepatitis B surface antigen and/or hepatitis C antibody and/or a known history of chronic hepatitis B or chronic hepatitis C were excluded. Patients with a known history of autoimmune liver diseases, or any other chronic liver diseases were also excluded. In addition, we excluded patients with significant alcohol consumption defined by >30 g/day in men and >20 g/day in women. The epidemiological, clinical data and outcomes were collected from medical records of patients.

The study was approved by the Ethics Committees of these hospitals, with a waiver of informed consent.

### 2.2 Definition of NAFLD, fibrosis, and severe illness

The NAFLD was diagnosed by the Hepatic Steatosis Index (HSI) in the absence of other causes of CLDs.^17^ The HSI was calculated by the equation: HSI = 8 × (ALT/AST ratio) + BMI (+2, if female; +2, if diabetes).^17^ Serum ALT and AST levels of the first test after patient admission were used for the calculation of the HSI. A cutoff of 36 of HSI was used to diagnose the presence of NAFLD.^18^, ^19^, ^20^ The HSI has been validated and used in previous studies.^18^, ^19^, ^20^

NAFLD fibrosis score was used to evaluate the severity of liver fibrosis.^15^ NFS was calculated by the following formula: NFS = −1.675 + 0.037 × age (year) +0.094 × BMI (kg/m^2^) +1.13 × IFG/diabetes (yes = 1, no = 0) + 0.99 × AST/ALT ratio − 0.013 × platelet count (×10^9^/L) ‐ 0.66 × albumin(g/dL).^15^ Patients were separated into two groups: advanced liver fibrosis (ALF) and non‐advanced liver fibrosis (non‐ALF) according to NFS.^15^, ^21^ Patients with NFS < −1.5 were defined as non‐ALF, while patients with the NFS at least −1.5 were defined as ALF.^15^

Severe illness of COVID‐19 was defined as COVID‐19 patients who fulfilled any of the following criteria: (1) respiratory frequency ≥ 30/min, (2) pulse oximeter oxygen saturation ≤ 93% at rest, and (3) oxygenation index ≤ 300 mmHg.^22^

### 2.3 Statistical analysis

Data were analyzed by SPSS version 22.0 software (SPSS Inc., Chicago, IL, United States). Continuous data were shown as medians and interquartile range (IQR), while categorical data were presented as the counts and percentages. Two‐sample t tests or Mann‐Whitney U test were used to compare the continuous variables between two groups as appropriate, while chi‐square tests or Fisher's exact tests were used to compare the categorical variables. Logistic regression analysis was used to identify the risk factors of ALF. Variables having *p* values < 0.1 in the univariate analysis were used for a multivariate input logistic regression analysis. Age and gender were also adjusted by the multivariate logistic regression analysis. *p* < 0.05 was considered statistically significant.

## 3 RESULTS

### 3.1 Demographic characteristics and onset symptoms

Among 342 screened patients with COVID‐19, 34 patients lacking BMI data, 7 patients with insufficient biochemistry data, 7 patients with alcohol abuse, and 14 patients concomitant with other chronic liver diseases (12 chronic hepatitis B, 1 chronic hepatitis C, and 1 Dubin‐Johnson syndrome) were excluded. 194 COVID‐19 patients without NAFLD identified by HSI were also excluded. Finally, 86 COVID‐19 patients with NAFLD were included in this study. Among the 86 patients consisting of 38 patients with ALF, the median age was 43.5 (IQR, 32.8–53.3) years and 58.1% were male. Patients with ALF were older (median 52.0 vs. 36.5 years, *p* < 0.001). More NAFLD patients with ALF had obese (63.2% vs. 22.9%, *p* < 0.001) and diabetes (23.7% vs. 2.1%, *p* = 0.006). More NAFLD patients with ALF had fever (81.6% vs. 50.0%, *p* = 0.002) and shortness of breath (18.4% vs. 0, *p* = 0.007). Moreover, the proportion of patients with severe illness was also significantly higher in the ALF compared to patients with non‐ALF (28.9% vs. 2.1%, *p* < 0.001) (Table 1).

**TABLE 1:** Demographic data and clinical outcomes of COVID‐19 patients who have NAFLD with or without advanced liver fibrosis

Variables (*n* [%] or median [IQR])	Non‐ALF(*n* = 48)	ALF(*n* = 38)	*p* value
Age (years)	36.5 (29.0,47.0)	52.0 (43.3,63.0)	<0.001
Age range			
≤60	46 (95.8)	28 (73.7)	0.003
>60	2 (4.2)	10 (26.3)	
Gender			
Male	29 (60.4)	21 (55.3)	0.630
Female	19 (39.6)	17 (44.7)	
BMI (kg/m^2^)	25.8 (24.5,27.7)	28.9 (26.8,30.2)	<0.001
BMI range			
<28	37 (77.1)	14 (36.8)	<0.001
≥28	11 (22.9)	24 (63.2)	
Onset signs and symptoms			
Fever	24 (50.0)	31 (81.6)	0.002
Cough	25 (52.1)	22 (57.9)	0.591
Fatigue	8 (16.7)	8 (21.1)	0.604
Sore throat	6 (12.5)	4 (10.5)	1.000
Muscle ache	4 (8.3)	3 (7.9)	1.000
Shortness of breath	0	7 (18.4)	0.007
Headache	2 (4.2)	1 (2.6)	1.000
Comorbidities			
Any comorbidity^a^	9 (18.8)	18 (47.4)	0.005
Hypertension	6 (12.5)	12 (31.6)	0.031
Diabetes	1 (2.1)	9 (23.7)	0.006
Chronic lung diseases	2 (4.2)	0	0.124
Outcomes			
Severe illness	1 (2.1)	11 (28.9)	<0.001
Admission to ICU	1 (2.1)	4 (10.5)	0.231
Death	0	0	

Abbreviations: ALF, advanced liver fibrosis; BMI, body mass index; ICU, intensive care unit; IQR, interquartile range.

^a^ Any comorbidity denoted for patients accompanied with one or more chronic diseases, such as hypertension, diabetes, and chronic lung diseases.

### 3.2 Laboratory and radiology examination

The baseline lymphocyte counts, platelet counts, and albumin levels were significantly lower in NAFLD patients with ALF compared with patients with non‐ALF (all *p* < 0.001). All NAFLD patients with ALF had pneumonia in chest CT and most (92.1%) of the patients had bilateral pneumonia. However, 12.5% of the NALFD patients with non‐ALF presented no pneumonia in chest CT. The proportion of pneumonia was higher in NAFLD patients with ALF than patients with non‐ALF (*p* = 0.028) (Table 2).

**TABLE 2:** Baseline laboratory parameters and chest CT of COVID‐19 patients who have NAFLD with or without advanced liver fibrosis

Variables (*n* [%] or median [IQR])	Non‐ALF(*n* = 48)	ALF(*n* = 38)	*p* value
WBC (×10^9^/L)	6.1 (4.9,6.9)	5.1 (3.9,6.7)	0.086
Lymphocytes (×10^9^/L)	1.6 (1.2,2.2)	1.1 (0.8,1.6)	<0.001
PLT (×10^9^/L)	227.5 (197.0,276.0)	152.0 (124.0,178.5)	<0.001
ALT (U/L)	36.5 (25.3,48.5)	33.5 (24.8,55.3)	0.955
AST (U/L)	23.4 (19.0,27.8)	31.0 (23.8,43.5)	<0.001
GGT (U/L)	32.5 (16.2,48.0)	36.7 (27.8,52.9)	0.095
Tbil (μmol/L)	11.3 (7.9,16.2)	10.7 (7.7,17.5)	0.920
ALB (g/L)	42.8 (39.1,46.7)	38.0 (34.9,41.4)	<0.001
ALP (U/L)	65.0 (51.0,73.0)	62.0 (51.5,72.8)	0.733
FBG (mmol/L)	5.2 (4.8,5.9)	6.5 (5.5,8.0)	<0.001
TG (mmol/L)	1.4 (1.0,2.3)	1.4 (1.0,1.5)	0.404
TC (moll/L)	3.9 (3.3,4.6)	4.0 (3.4,4.7)	0.641
Chest CT			
No pneumonia	6 (12.5)	0	0.028
Unilateral pneumonia	8 (16.7)	3 (7.9)	
Bilateral pneumonia	34 (70.8)	35 (92.1)	

Abbreviations: ALB, albumin; ALP, alkaline phosphatase; ALT, alanine transaminase; AST, aspartate aminotransferase; GGT, gamma‐glutamyl transferase; NAFLD, non‐alcoholic fatty liver disease; PLT, platelet; Tbil, total bilirubin.

### 3.3 Risk factors of severe illness during hospitalization

Logistic regression analysis was performed to identify the risk factors of severe illness in COVID‐19 patients. The univariate analysis revealed that obese (BMI ≥ 28 kg/m^2^) (odds ratio [OR] 5.538, 95% confidence interval [CI] 1.378–22.260, *p* = 0.016), concurrent diabetes (OR 9.857, 95% CI 2.282–42.582, *p* = 0.002), and ALF (OR 19.148, 95% CI 2.342–156.542, *p* = 0.006) were associated with severe illness. Multivariate analysis indicated that concurrent diabetes (OR 8.264, 95% CI 1.202–56.830, *p* = 0.032) and ALF (OR 11.057, 95% CI 1.193–102.439, *p* = 0.034) were independent risk factors of severe illness in COVID‐19 patients with NAFLD (Table 3).

**TABLE 3:** Risk factors of severe illness during hospitalization in COVID‐19 patients

Variables	Univariate		Multivariate	
	OR (95%CI)	*p* value	OR (95%CI)	*p* value
Age (years)				
≤60	Reference			
>60	1.280 (0.244,6.719)	0.770	4.891 (0.538,44.451)	0.159
Gender				
Female	Reference			
Male	1.009 (0.293,3.478)	0.988	0.698 (0.150,3.239)	0.646
BMI (kg/m^2^)				
<28	Reference			
≥28	5.538 (1.378,22.260)	0.016	3.320 (0.644,17.103)	3.320
Hypertension				
No	Reference			
Yes	0.305 (0.037,2.533)	0.271		
Diabetes				
No	Reference			
Yes	9.857 (2.282,42.582)	0.002	8.264 (1.202,56.830)	0.032
Advanced liver fibrosis				
No	Reference			
Yes	19.148 (2.342,156.542)	0.006	11.057 (1.193,102.439)	0.034

Abbreviations: BMI, body mass index; CI, confidence interval; ICU, intensive care unit; OR, odd ratio.

## 4 DISCUSSION

The presence of NAFLD has been reported as a predictor of the development of liver injury.^11^, ^12^ In our previous study, we also demonstrated that COVID‐19 patients with NAFLD are more likely to develop liver injury.^10^ However, the association of liver fibrosis and the severity of COVID‐19 is not yet clear. Forlano et al reported that the presence of intermediate/high‐risk FIB‐4 or liver cirrhosis was not associated with ICU admission and in‐hospital mortality of NAFLD patients with COVID‐19.^23^ However, the sample size is very small which only included 38 patients.^23^ Targher et al reported that the severity of COVID‐19 was associated with intermediate (unadjusted OR 4.32) or high (unadjusted OR 5.73) FIB‐4 scores in patients with MAFLD.^14^ The non‐invasive fibrosis scores such as FIB‐4 and NFS have been shown to predict fibrosis stage of NAFLD patients with reasonable accuracy.^24^, ^25^ NFS score was initially developed to evaluate liver fibrosis degree in NAFLD patients.^15^ A prospective study demonstrated that NFS had a more excellent discriminatory ability for the detection of advanced fibrosis than FIB‐4.^26^ Another study also reported that NFS had the highest discriminatory accuracy for advanced fibrosis compared with FIB‐4.^27^ However, there was no study investigating the association with NFS and the severity of COVID‐19 in patients with NAFLD. In this study, we found that 44.2% of the NAFLD patients with COVID‐19 had ALF according the NFS. Furthermore, we found that COVID‐19 patients with NAFLD and increased NFS was an independent risk factor of severe illness of patients with COVID‐19. We also found that the presence of diabetes was an independent risk factor of severe illness of COVID‐19 which is consistent with the previous study.^28^

The exact pathophysiology on how ALF contributes to a more severe COVID‐19 illness in NAFLD patients is not yet clear, while several potential mechanisms have been proposed. Systemic inflammatory response syndrome, especially the “cytokine storm,” is one of the important pathophysiologic mechanisms for the development of severe illness in COVID‐19.^29^ The presence of ALF might exacerbate the virus‐induced “cytokine storm” and thereby contribute to severe COVID‐19 through the hepatic release of proinflammatory cytokines.^14^ However, more studies are needed to explore the mechanisms of advanced NAFLD contributing to the COVID‐19 disease process.

Our study has some limitations. First, the sample size of our study is relatively small. Second, we use the non‐invasive fibrosis scores to diagnose fibrosis without a histological diagnosis of liver fibrosis. Other non‐invasive tests such as transient elastography by FibroScan may be more reliable for the liver fibrosis and steatosis assessment.^30^ However, during the early stages of the COVID‐19 outbreak, the transient elastography was not performed in the patients. Third, we did not analyze the risk factors of ICU admission of COVID‐19 patients with NAFLD since only 5 patients were admitted to the ICU. Fourth, given that the quality of care may vary across hospitals, the results might be biased by the heterogeneity of care across hospitals. However, all patients were treated according to the guidelines for the Diagnosis and Treatment of coronavirus disease 2019 (COVID‐19) issued by the National Health Commission of China.^22^ Thus, we consider the quality of care across hospitals might not significantly impact our results.

Our study demonstrates that patients with NAFLD with NFS‐determined ALF are at higher risk of having severe COVID‐19. However, more studies with large sample size are needed to validate our results and to better understand the mechanistic link of advanced NAFLD and severe COVID‐19.

## CONFLICTS OF INTEREST

The authors have declared that no conflicts of interest exist.

## AUTHORS’ CONTRIBUTIONS

Concept and design: Chao Wu, Rui Huang and Ming Li. Drafting of the manuscript: Renling Yao, Rui Huang, Jian Wang, Li Zhu, Jiacheng Liu; Critical revision of the manuscript for important intellectual content: Chuanwu Zhu and Ming Li; Statistical analysis: Jian Wang and Jiacheng Liu; Administrative, technical, or material support: Longgen Liu, Haiyan Zhao; Supervision: Ming Li, Chao Wu and Rui Huang; Acquisition, analysis, or interpretation of data: Renling Yao, Rui Huang, Jian Wang, Jiacheng Liu, Ruifei Xue, Leyang Xue, Songping Huang, Xiang‐an Zhao, Chunyang Li, Yang Li and Juan Cheng.

## Data Availability

The data that support the findings of this study are available on request from the corresponding author. The data are not publicly available due to privacy or ethical restrictions.
